# The supply-side climate policy of decreasing fossil fuel tax profiles: can subsidized reserves induce a green paradox?

**DOI:** 10.1007/s10584-022-03389-w

**Published:** 2022-08-22

**Authors:** Garth Day, Creina Day

**Affiliations:** Australian National University, Canberra, Australia

**Keywords:** Supply-side climate policy, Fossil fuel producer subsidy, Taxation

## Abstract

Fossil fuel producers develop too many reserves for combustion due to subsidies for upfront development costs. The conventional wisdom is that downward-sloping tax profiles avoid green paradox outcomes by reducing present extraction. This paper shows that accounting for subsidized reserves development can induce green paradox outcomes for downward-sloping income tax profiles. A theoretical model linking reserves development and extraction with climate change damages is developed to explore conditions for the weak and strong green paradox outcomes of higher present extraction and cumulative damages. We find that the weak green paradox arises under higher and flatter income tax profiles. The strong green paradox is an ambiguous outcome without subsidized reserves development. Quantitative examples demonstrate the effect of downward-sloping tax profiles on crude oil extraction and how the strong green paradox arises when delayed emissions are less relevant for damages.

**Supplementary Information:**

The online version contains supplementary material available at 10.1007/s10584-022-03389-w.

## Introduction

Fossil fuel producers hold reserves which are exhaustible, yet excessive in terms of the Paris agreement to limit global warming well below 2 °C and pursue efforts to limit the temperature increase to 1.5 °C relative to pre-industrial times. A contemporary challenge for climate policy is how to curtail the development and extraction of fossil fuel reserves. Resource owners have an incentive to bring forward extraction if they anticipate tightening of climate policy. Accelerated fossil fuel combustion and carbon emissions exacerbate climate change damages. The existing literature has shown that downward-sloping tax profiles provide one mechanism to reduce present extraction whereby policymakers can avoid green paradox outcomes. This paper explores whether accounting for subsidized reserves development in the form of tax deductions for upfront costs can induce green paradox outcomes for a downward-sloping income tax profile.

Existing fossil fuel reserves if used in their entirety would generate three times more carbon dioxide emissions than recommended to keep the global temperature rise under 2 °C (International Energy Agency 2020). By 2050, nearly 60% of the world’s oil reserves, 84% of Canada’s oil sands, and virtually all coal reserves in Australia and Russia must be left in the ground if global warming is to be kept below 1.5 °C (Welsby et al. 2021). These estimates disregard new reserves in the offing. One-third of potential supply from new oil projects would be unneeded under the 2 °C target (Carbon Tracker 2017). In fact, new oil and gas fields and new coal mines and mine extensions must cease if the 1.5 °C and zero emissions by 2050 targets are to be met (International Energy Agency 2021).

Fossil fuel producer subsidies in the order of $70 billion on average annually for G20 nations in 2013 and 2014 (Bast et al. 2015) are less than consumer subsidies, but nonetheless significant. The timing of producer subsidies through tax deductions for exploration and infrastructure costs distorts decisions to bring on new supply. Tax preferences push nearly half of new oil, yet to be developed as reserves, into profitability (Erickson et al. 2017). Cancelation of accelerated depreciation for new capital investment alone would account for 10% and 13% of the reduction in global emissions from producer subsidy removal in the USA (Erickson et al. 2020) and Canada, Norway, and Russia (Jewell et al. 2020), respectively.

The COVID-19 pandemic triggered a historic drop in oil demand for 2020 and has prompted downward revisions in long-term forecasts for growth in demand and prices. Jaccard et al. (2018) find that a less than 5% probability of viable Canadian oil sands expansion under the 2 °C carbon budget rises to 30% if OPEC agrees to significantly reduce its market share by 2045. However, existing modeling of fossil fuel supply and climate change overlooks the effect of subsidized reserves development costs and intertemporal changes in demand. The model developed here incorporates these themes.

This paper contributes to an emerging literature on supply-side policies that aim to curtail the size and extraction of fossil fuel reserves. Supply-side measures include government compensation for leaving fossil fuel undeveloped (Harstad 2012; Eichner and Pethig 2019), depletion quotas and confiscation of reserves (Asheim 2013), taxation of production (Faehn et al. 2017) and coalition of coal producers (Richter et al. 2018), limits on exploration and the removal of producer subsidies (Lazarus and van Asselt 2018), and ceasing the issuance of new leases for fossil fuel extraction (Erickson and Lazarus 2018). However, Intergovernmental Panel on Climate Change reports and nationally determined contributions feature few if any supply-side measures (Piggot et al. 2018).

Whereas fossil fuel producing countries may oppose demand-side agreements that suppress prices, supply-side climate policy offers important economic and political benefits (Green and Denniss 2018). Curbing the volume extracted for export has a positive terms of trade effect, whereby selling extracted fossil fuel at higher prices compensates producers for foregone income from unextracted reserves (Asheim et al. 2019). Combining supply-side measures with demand-side measures provides clearer signals on the future of fossil fuel production to discourage infrastructure investment (Erickson et al. 2015) and lower overall costs of achieving emission targets, by one-third in the case of Norway (Faehn et al. 2017). Shifting the carbon tax base from emissions to fossil fuel supply allows producers to retain a higher share of fossil fuel rents for reinvestment in low carbon assets (Peszko et al. 2020).

Well-intentioned policy may enhance rather than mitigate climate change by distorting the intertemporal supply of exhaustible fossil fuels. First coined by Sinn (2008, 2012, 2015), the green paradox occurs when fossil fuel producers anticipate that tightening of climate policy dampens future prices and accelerate extraction for fear of being stranded with worthless polluting assets. This in turn worsens climate change damages where present emissions are more harmful than future emissions.

Existing modeling of the green paradox focuses on demand-side policy. Producers bring forward extraction and emissions in response to the development of low carbon energy substitutes, known in the literature as backstop technology (Strand 2007; Hoel 2008). The green paradox may vanish when backstop technology is sufficiently cheap relative to emissions damages that it pays to leave reserves unextracted (van der Ploeg and Withagen 2012). The increasing marginal cost of renewable energy and extraction counters a weak and strong green paradox, defined as higher present emissions which are assumed proportional to extracted fossil fuel, and higher net present value of cumulative damages from climate change, respectively (Gerlagh 2011). We find distinguishing weak and strong green paradox outcomes to be a useful approach for the analysis here, although our focus on the combination of subsidized reserves development and a downward-sloping income tax profile is different.

The consensus in the theoretical literature is that downward-sloping tax profiles avoid green paradox outcomes. Seminal models incorporate the result of Dasgupta and Heal (1979) that a downward-sloping profile for an ad valorem tax rate encourages slower extraction for given reserves of an exhaustible resource which is costless to extract. Sinn (2008) and Osterle (2015) show that an upward-sloping tax profile encourages accelerated extraction and global warming unless costs increase with the cumulative stock extracted faster than the tax rate rises. Sinclair (1994) models global warming as a stock externality where productivity increases with fossil fuel reserves left underground and finds that the socially optimal carbon tax rate has a downward-sloping profile under costless extraction. Allowing for constant extraction costs and decay of atmospheric carbon dioxide, Ulph and Ulph (1994) show that the socially optimal carbon tax rate declines as the climate system slowly returns to a natural state. While useful implications can be obtained for international agreements on demand-side climate policy, the roles of fossil fuel producer subsidy and taxation in supply-side climate policy are overlooked. This paper seeks to fill this gap in the literature.

The model in this paper links the economics of exhaustible reserves and extraction to climate change damages from fossil fuel extracted for sale and combustion. The profit-maximizing producer chooses how much fossil fuel to develop as extraction-ready reserves and when to extract for sale over time. The cost of reserves development is increasing in the size of reserves and subsidized through deductions at the initial income tax rate. Fossil fuel demand is decreasing in the price which the producer takes as given. Earlier emissions cause higher net present value of climate change damages than delayed emissions from deferred extraction for sale and combustion.

In contrast to the existing literature, we find that accounting for reserves development subsidies can introduce a weak and strong green paradox for downward-sloping income tax profiles. The mechanism whereby future relative to present producer prices influence the timing of extraction and emissions relates to existing models. However, the analysis here recognizes how the level and downward slope of income tax profiles influence the size of reserves. Whereas tax rate levels do not distort present relative to future producer prices when reserves are fixed in existing models, we allow upfront deductions for reserves development costs to increase with the tax rate level. Moreover, lower future tax rates have an ambiguous effect on the present volume of extraction due to competing incentives to develop more reserves and delay extraction.

Two particularly interesting results of the analysis are, firstly, that the weak green paradox arises under a higher level and flatter intertemporal profile for the income tax rate. Secondly, whereas the weak green paradox vanishes for any downward-sloping income tax profile without the producer subsidy, the strong green paradox arises when delayed emissions are more relevant for climate damages. These findings challenge the conventional wisdom that downward-sloping tax profiles avoid green paradox outcomes irrespective of the tax rate level.

The intuition for the core results lies in scale and intertemporal substitution effects. Upfront tax deductions reduce the incremental cost relative to the value of scaling up development of fossil fuel reserves. Lower future tax rates confer an incentive both to develop more reserves and delay extraction to the future. The higher the level of income tax rate, the higher the upfront tax deduction which strengthens the former effect. Flatter income tax profiles counter the latter effect. The overall volume of fossil fuel extracted for current sale and combustion increases, which exacerbates climate change damages where present emissions are more harmful than future emissions.

## Model of fossil fuel reserves and extraction

Consider a producer with an exhaustible stock of fossil fuel, ${S}_{t}$, that depletes over time $t$ according to

1 $$\begin{array}{c}\begin{array}{ccc}\dot{S}=-{E}_{t};& {S}_{t}\ge 0;& {S}_{0}>0\end{array} endogenous\end{array}$$

where ${S}_{0}$ denotes the endogenous stock of reserves and ${E}_{t}$ denotes the volume of resources extracted for sale at time $t$. Extraction activity commences when resources are accessible at initial time $t=0$.

The convex cost of fossil fuel development is

2 $$\begin{array}{c}\Phi \begin{array}{ccc}\left({S}_{0}\right);& {\Phi }^{^{\prime}}>0,& {\Phi }^{^{\prime\prime} }>0\end{array}\end{array}$$

where the marginal cost of developing reserves to the point where they can be extracted increases as the stock of extraction-ready reserves increases. This is consistent with the rising marginal cost of exploration and development, especially for non-conventional fossil fuel, such as shale oil and tar sands.

The inverse demand curve for fossil fuel is

3 $$\begin{array}{c}\begin{array}{ccc}{p}_{t}=p\left({E}_{t},{Z}_{t}\right);& \frac{\partial {p}_{t}}{\partial {E}_{t}}<0,& \frac{\partial {p}_{t}}{\partial {Z}_{t}}>0\end{array}\end{array}$$

where the seller takes prices ${p}_{t}$ as given. The direct demand curve ${E}_{t}=E\left({p}_{t},{Z}_{t}\right)$ is decreasing and concave in prices $\left({E}^{^{\prime}}<0; {E}^{^{\prime\prime} }>0\right)$ where ${Z}_{t}$ is a shift parameter. To allow for shifts in fossil fuel demand over time, let $\dot Z/Z=\pi\gtreqless0$ denote the intertemporal rate of change in demand.

The income tax rate ${\tau }_{t}$ may vary over time according to

4 $$\begin{array}{c}\begin{array}{cc}\left(1-{\tau }_{t}\right)=\left(1-{\tau }_{0}\right){e}^{xt};& {\tau }_{t}\in \left(\mathrm{0,1}\right)\end{array}\end{array}$$

where ${\tau }_{0}$ is the tax rate at $t=0$ and $x=-{\dot{\tau }}_{t}/\left(1-{\tau }_{t}\right)>0$ is the growth rate in $\left(1-{\tau }_{t}\right)$. The income tax rate rises (falls) over time when $x<(>)0$. Tax deductions claimed for the cost of fossil fuel development are $\delta {\tau }_{0}\Phi \left({S}_{0}\right)$, where $\delta >1$ reflects a fossil fuel producer subsidy.^1^

The net present value of total profits equals the after-tax revenue stream minus the upfront cost of reserves net of tax deductions. As in Sinclair (1994) and Dasgupta and Heal (1979), zero marginal extraction cost is assumed at any time $t$ for the purpose of focusing the analysis on the effect of the timing of producer subsidy through income tax deductions. Profit maximization comprises two decisions: first, the size of total reserves to be extracted; second, the scheduling of extraction for sale over time.

Formally, the producer chooses ${S}_{0}$ and ${E}_{t}$ to maximize

5 $$\begin{array}{c}{\int }_{0}^{\infty }\left(1-{\tau }_{0}\right){e}^{xt}{p}_{t}{E}_{t}{e}^{-rt}dt-\left(1-\delta {\tau }_{0}\right)\Phi \left({S}_{0}\right)\end{array}$$

subject to (1) where future revenue is discounted at the rate $r$. Referring to the appendix,^2^ the dynamic optimization problem gives three conditions which determine the supply of fossil fuel.

The static efficiency condition for fossil fuel extraction is

6 $$\begin{array}{c}\left(1-{\tau }_{t}\right)p\left({E}_{t},{Z}_{t}\right)={\mu }_{t}\end{array}$$

where ${\mu }_{t}$ is the shadow price of unextracted fossil fuel at time $t$ and $\left(1-{\tau }_{t}\right)p\left({E}_{t},{Z}_{t}\right)$ is the after-tax marginal revenue from resource sales at time $t$. The marginal value of unextracted fossil fuel equals the after-tax incremental revenue from an additional unit extracted for sale at any point in time.

The condition for profit-maximizing fossil fuel reserves is

7 $$\begin{array}{c}\left(1-\delta {\tau }_{0}\right){\Phi }^{^{\prime}}\left({S}_{0}\right)={\mu }_{0}\end{array}$$

where ${\Phi }^{^{\prime}}\left({S}_{0}\right)$ is the marginal cost of developing recoverable fossil fuel at $t=0$ and ${\mu }_{0}$ is the shadow price of unextracted fossil fuel at $t=0$. The producer chooses the size of reserves to equate the net incremental cost of developing an additional unit of reserves with the incremental value of an additional unit of reserves at the time extraction activity commences.

Equations (4) and (6) imply the condition for dynamic efficiency

8 $$\begin{array}{c}x+\frac{\dot{p}}{p}=r\end{array}$$

which says that efficient intertemporal management of fossil fuel equates the rate of return to holding unextracted resources and capital. This is the tax-adjusted Hotelling rule where the rate of return to leaving resources in the ground equals the growth rate in the amount that could be earned from selling an additional unit due to marginal revenue increasing at rate $\dot{p}/p>0$ and the tax rate on revenue decreasing at rate $x>0$. Extraction of fossil fuel for sale is scheduled so that discounted after-tax marginal revenue remains constant over time. If the equality in (8) did not hold, then the mining firm could increase discounted profits by adjusting extraction ${E}_{t}$ until returns to unextracted resources and capital were equalized.

For an explicit solution, consider the demand function

9 $$\begin{array}{c}{E}_{t}={{Z}_{t}\left({p}_{t}\right)}^{-\eta }\end{array}$$

with constant price elasticity $\eta =-\frac{d{E}_{t}}{{E}_{t}}/\frac{d{p}_{t}}{{p}_{t}}$ and the cost of reserves function

10 $$\begin{array}{c}\Phi \left({S}_{0}\right)=\varphi \frac{{{S}_{0}}^{1+\alpha }}{1+\alpha }\end{array}$$

where $\varphi >0$ and $\alpha >0$. Substituting from (9) for $\dot{p}/p=\frac{1}{\eta }\left(\pi -\dot{E}/E\right)$ in (8), the rate of change in volume of resources extracted is

11 $$\begin{array}{c}\frac{\dot{E}}{E}=-\left[\eta \left(r-x\right)-\pi \right]\end{array}$$

where $r>\pi /\eta +x$ implies that the time path of extracted fossil fuel is downward sloping where ${E}_{t}$ satisfies constraint (1). The rationale for (11) lies in the tax-adjusted Hotelling rule. Intuitively, fossil fuel is extracted over time at a rate that leaves the producer indifferent between extracting an additional unit for sale and leaving it in the ground to appreciate in value.

The extraction rate $\eta \left(r-x\right)-\pi$ is less than $\eta r$ if there are outward shifts in demand over time $\left(\pi >0\right)$ and the income tax profile is downward sloping $\left(x>0\right)$ because the higher return to unextracted fossil fuel confers an incentive to postpone extraction to a later date. The extraction rate is decreasing in the rate at which the producer discounts revenue $r$ because lower discounted future marginal revenue confers an incentive to extract at an earlier date.

Equation (11) implies that a downward trend in the income tax rate flattens the time path of fossil fuel extracted for sale. We refer to the effect on the slope of the extraction time path as the *intertemporal substitution effect*. For given fossil fuel reserves and demand trends, lower tax rates on income in the future encourage the fossil fuel producer to defer extraction to the future. However, whether the volume of fossil fuel left unextracted increases depends on the effect of the tax system on reserves. While the dynamic efficiency condition (8) gives the *slope* of the extraction time path, the conditions for static efficiency (6) and reserves of fossil fuel (7) give *present* and *aggregate* fossil fuel extracted along the time path.

Referring to the appendix, the time paths for the fossil fuel shadow value, price, and extraction are

12 $$\begin{array}{c}{\mu }_{t}=\left(1-{\tau }_{0}\right){\left(\frac{{Z}_{0}}{{S}_{0}\left(\eta \left(r-x\right)-\pi \right)}\right)}^{\frac{1}{\eta }}{e}^{rt}\end{array}$$

13 $$\begin{array}{c}{p}_{t}={\left(\frac{{Z}_{0}}{{S}_{0}\left(\eta \left(r-x\right)-\pi \right)}\right)}^{\frac{1}{\eta }}{e}^{\left(r-x\right)t}\end{array}$$

14 $$\begin{array}{c}{E}_{t}={{S}_{0}\left(\eta \left(r-x\right)-\pi \right)e}^{-\left(\eta \left(r-x\right)-\pi \right)t}\end{array}$$

where $r>x$ and fossil fuel reserves are

15 $$\begin{array}{c}{S}_{0}={\left[\frac{\left(1-{\tau }_{0}\right)}{\left(1-\delta {\tau }_{0}\right)\varphi }{\left(\frac{{Z}_{0}}{\eta \left(r-x\right)-\pi }\right)}^{\frac{1}{\eta }}\right]}^{\frac{\eta }{1+\alpha \eta }}\end{array}$$

which is increasing in the shift parameter for level of demand $\left({Z}_{0}\right)$ relative to the shift parameter for level of marginal cost of reserves development $\left(\varphi \right)$, outward shifting demand over time $\left(\pi >0\right)$, the rate of decline in the income tax rate $\left(x>0\right)$, and the initial income tax rate $\left({\tau }_{0}\right)$ when the cost of reserves is subsidized $\left(\delta >1\right)$. The rationale for (15) lies in the conditions for static efficiency and reserves of fossil fuel. Intuitively, the profit-maximizing size of reserves equates the incremental value of unextracted fossil fuel with the after-subsidy incremental cost of developing an additional unit of reserves and the after-tax incremental revenue from an additional unit extracted for sale.

Equation (15) says that a downward trend in the income tax rate increases extractable reserves of fossil fuel. We refer to the effect on the size of fossil fuel reserves as the *scale effect*. All else equal, lower tax rates on income in the future increase the value of fossil fuel in the ground which encourages the producer to develop more reserves.

## Climate change damages and the green paradox

Taking the lead of Gerlagh (2011), climate change damages are cumulative and captured through the function $D\left({E}_{t}\right)={\int }_{0}^{\infty }{e}^{-rt}{\theta }_{t}{E}_{t}dt$ with a shadow price on emissions ${\theta }_{t}={\theta }_{0}{e}^{\sigma t}$ which grows at rate $\sigma \in \left(\mathrm{0,1}\right)$ over time. Global greenhouse gas emissions increase proportional to the flow of fossil fuel extracted for consumption, ${E}_{t}$. The increasing shadow price is consistent with marginal damages per emissions increasing over time due to increasing greenhouse gas concentrations in the atmosphere (Ulph and Ulph 1994; Hoel and Kverndokk 1996).

The net present value (NPV) of climate change damages is

16 $$\begin{array}{c}D\left({E}_{t}\right)={\int }_{0}^{\infty }{e}^{-\rho t}{\theta }_{0}{E}_{t}dt\end{array}$$

where $\rho =r-\sigma$ and the assumption $\rho \in \left(\mathrm{0,1}\right)$ means that marginal damages per emissions grow at a slower rate than the shadow price of unextracted fossil fuel. This assumption is consistent with a carbon cycle where natural depreciation of greenhouses gases, albeit small, lowers the rate of increase in the shadow price on emissions compared to the rate of increase in the shadow price of the absorption capacity which follows the Hotelling rule. In the context of the green paradox, the assumption implies that earlier emissions from fossil fuel extracted and burned cause higher NPV damages than delayed emissions from deferred extraction and combustion.

We now define the weak and strong green paradox for the policy scenario which comprises an upfront subsidy for the cost of reserves $\left(\delta >1\right)$ and income tax rate which declines over time $\left(x>0\right)$. The approach taken is to compare current emissions and NPV damages under the policy and laissez-faire scenarios.

### Definition

*A weak green paradox occurs when current extraction of fossil fuel for sale and thus emissions are higher under the producer subsidy and declining tax rate compared with laissez-faire extraction,* ${E}_{0}>{E}_{0}^{LF}$ *.*

### Definition

*A strong green paradox occurs when net present value of climate change damages is higher under the producer subsidy and declining tax rate compared with the laissez-faire outcome* *,* $D\left({E}_{t}\right)>D\left({E}_{t}^{LF}\right)$ *.*

### Weak green paradox

Referring to the appendix, a weak green paradox occurs if

17 $$\begin{array}{c}\frac{\left(1-{\tau }_{0}\right)}{\left(1-\delta {\tau }_{0}\right)}>{\left(\frac{\eta r-\pi }{\eta \left(r-x\right)-\pi }\right)}^{\alpha }\end{array}$$

where $\left(1-{\tau }_{0}\right)/\left(1-\delta {\tau }_{0}\right)>1$ when $\delta >1$ and $\left(\eta r-\pi \right)/\left(\eta \left(r-x\right)-\pi \right)>1$ when $x>0$. The reverse inequality may hold, in which case the weak green paradox is avoided. In the context of policy, (17) identifies values of ${\tau }_{0}$ and $x$ for which the weak green paradox is more likely to occur.

The inequality in (17) can be rewritten in terms of tax schedule parameters

18 $$\begin{array}{c}x<\left(r-\frac{\pi }{\eta }\right)\left[1-{\left(\frac{1-\delta {\tau }_{0}}{1-{\tau }_{0}}\right)}^{\frac{1}{\alpha }}\right]\end{array}$$

19 $$\begin{array}{c}{\tau }_{0}>\frac{{\left(\eta r-\pi \right)}^{\alpha }-{\left(\eta \left(r-x\right)-\pi \right)}^{\alpha }}{\delta {\left(\eta r-\pi \right)}^{\alpha }-{\left(\eta \left(r-x\right)-\pi \right)}^{\alpha }}\end{array}$$

which says that a weak green paradox arises for a sufficiently low intertemporal rate of decline and high initial level of the tax rate. This result is summarized in the following remark.

#### Remark 1

*The higher the initial income tax rate* $\left({\tau }_{0}^{H}\right)$ *and the lower the rate of decline in the tax rate *$\left({x}_{L}\right)$*, the more likely the weak green paradox.*

**Fig. 1:**
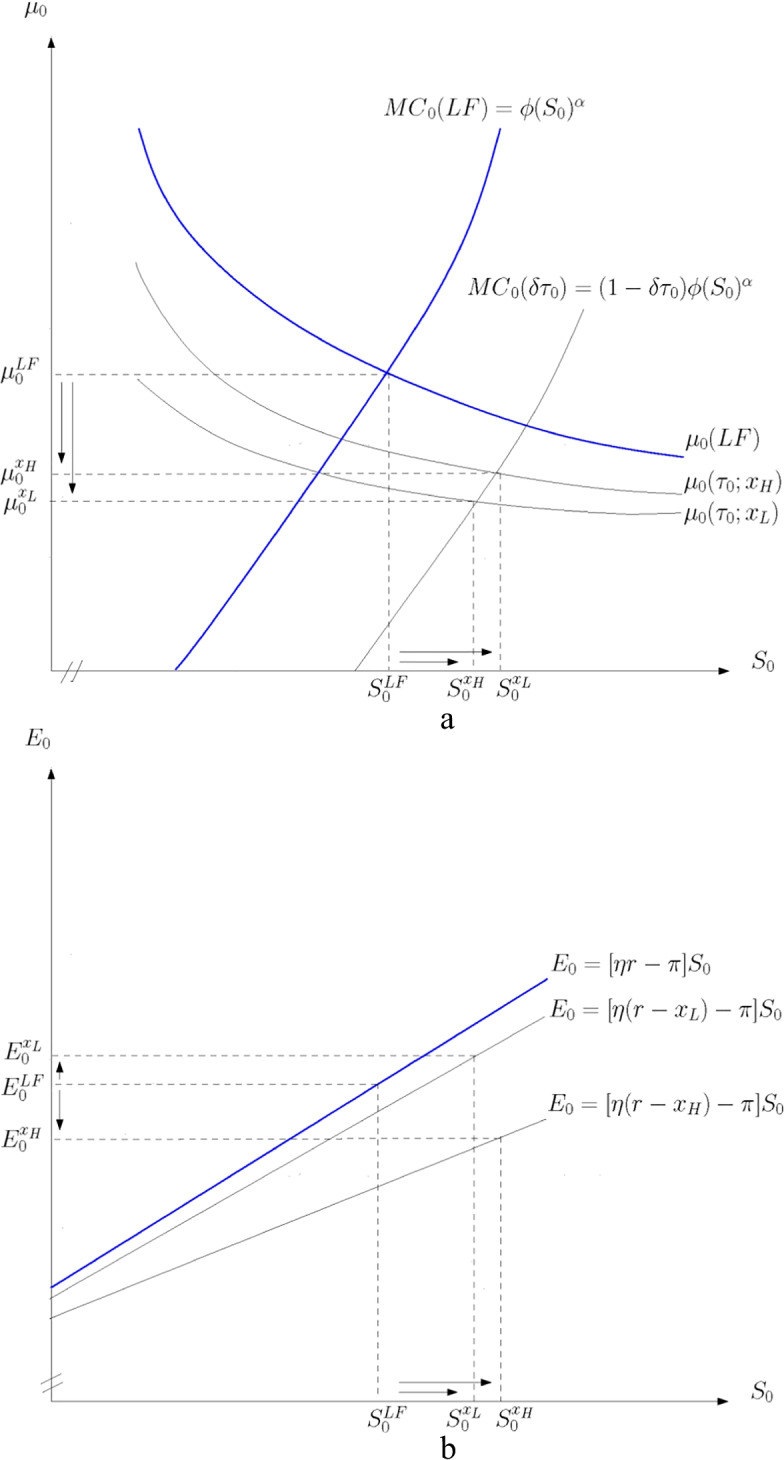
Effect of slope of income tax profile on reserves and extraction. **a** Reserves and rate of decline in the tax rate.** b** Extraction and rate of decline in the tax rate

Figure 1 illustrates the effect of low and high intertemporal rates of decline in the tax rate, ${x}_{L}$ and ${x}_{H}$. Figure 1a shows the effect on the profit-maximizing size of reserves ${S}_{0}$ where the private marginal cost and marginal value curves, given by (7) after substituting from (12) for a given ${S}_{0}$, intersect. The subsidized marginal cost $M{C}_{0}\left(\delta {\tau }_{0}\right)=\left(1-\delta {\tau }_{0}\right)\varphi {{S}_{0}}^{\alpha }$ and after-tax marginal value of reserves ${\mu }_{0}\left({\tau }_{0}; x\right)={\left(1-{\tau }_{0}\right)\left[{Z}_{0}/\left({S}_{0}\eta \left(r-x\right)-\pi \right)\right]}^{1/\eta }$ are lower than the laissez-faire marginal cost and marginal value of reserves, $M{C}_{0}\left(LF\right)=\varphi {{S}_{0}}^{\alpha }$ and ${\mu }_{0}\left(LF\right)={\left[{Z}_{0}/\left({S}_{0}\left(\eta r-\pi \right)\right)\right]}^{1/\eta }$. The subsidy reduces the marginal cost by more than the tax reduces the marginal value of reserves and thus ${S}_{0}$ increases relative to the laissez-faire outcome. The steeper the downward tax trend $\left({x}_{H}>{x}_{L}\right)$, the smaller the decrease in the initial shadow price of unextracted resources $\left({\mu }_{0}^{{x}_{L}}<{\mu }_{0}^{{x}_{H}}<{\mu }_{0}^{LF}\right)$ and the larger the increase in reserves $\left({S}_{0}^{{x}_{H}}>{S}_{0}^{{x}_{L}}>{S}_{0}^{LF}\right)$. Intuitively, lower future tax rates raise the return to holding unextracted resources and thus the profitability of developing additional reserves at higher incremental cost.

Figure 1b shows the effect on current extraction for sale ${E}_{0}$, given by (14). The decrease in the slope of the extraction line shows the negative intertemporal substitution effect. Lower future tax rates increase the growth rate in after-tax marginal revenue over time which encourages the fossil fuel producer to reschedule extraction of given reserves to the future. The movement rightward along the line due to ${S}_{0}>{S}_{0}^{LF}$ shows the positive scale effect. Lower future tax rates increase the marginal value of resources in the ground which encourages the producer to increase the size of reserves.

The intertemporal substitution effect shifts up the current price path as less current extraction of given reserves reduces current supply, while the scale effect moves down along the price path as developing more reserves increases supply. The lower (higher) the rate of decline in the tax rate, the weaker (stronger) the negative intertemporal substitution effect relative to the positive scale effect and thus, current extraction increases (decreases) relative to the laissez-faire scenario $\left({E}_{0}^{{x}_{L}}>{E}_{0}^{LF}>{E}_{0}^{{x}_{H}}\right)$. A weak green paradox therefore arises for ${x}_{L}$.

**Fig. 2:**
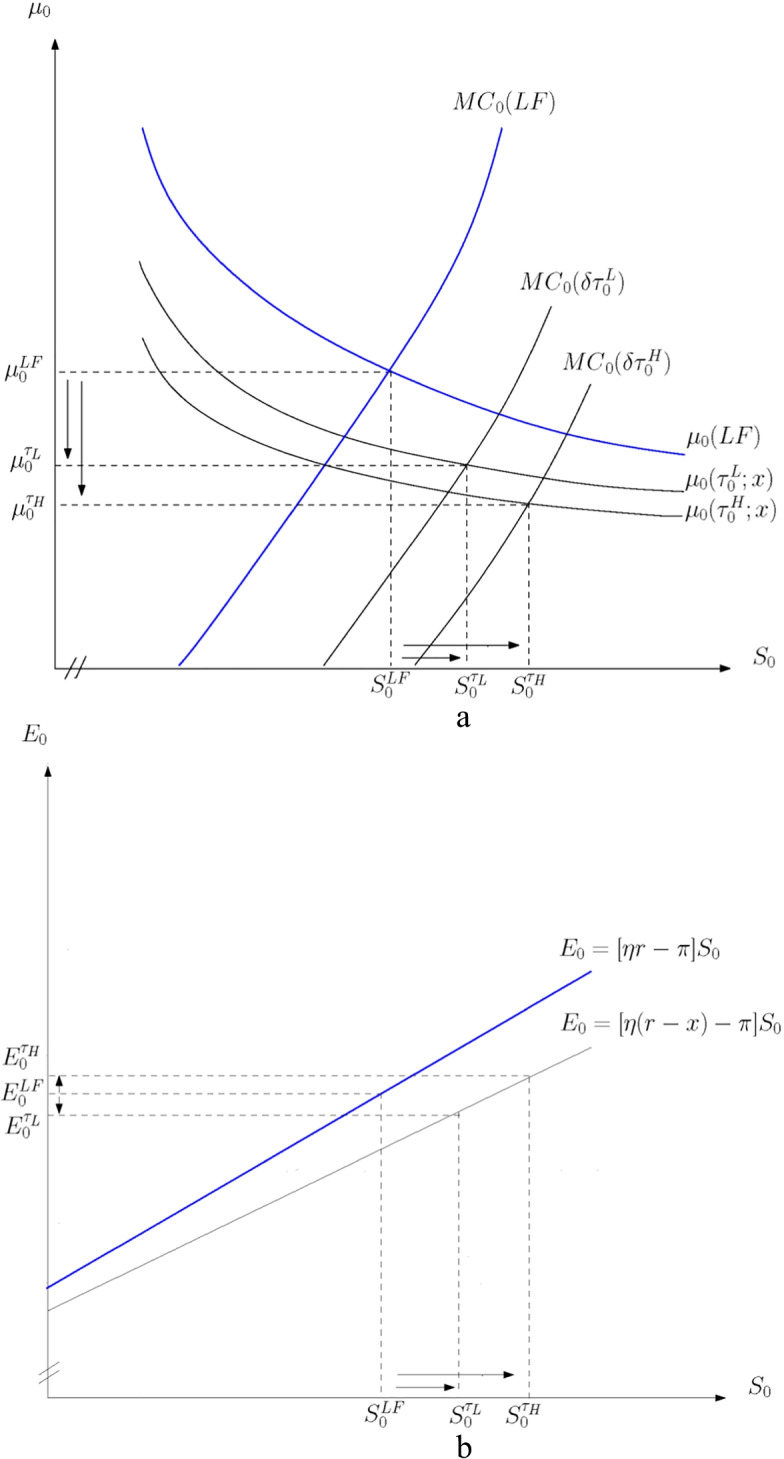
Effect of level of income tax profile on reserves and extraction. **a** Reserves and initial level of the tax rate. **b** Extraction and initial level of the tax rate

Figure 2 illustrates the effect of a low and high initial level of the tax rate, ${\tau }_{L}$ and ${\tau }_{H}$. Referring to Fig. 2a, the higher the level of the tax rate, the larger the decrease in the shadow price of unextracted resources $\left({\mu }_{0}^{{\tau }_{H}}<{\mu }_{0}^{{\tau }_{L}}<{\mu }_{0}^{LF}\right)$ and the larger the increase in profit-maximizing reserves $\left({S}_{0}^{{\tau }_{H}}>{S}_{0}^{{\tau }_{L}}>{S}_{0}^{LF}\right)$. The higher tax deduction for the cost of developing reserves and the lower after-tax discounted revenue stream reduce the shadow price of unextracted resources. The lower after-subsidy marginal cost of reserves relative to the after-tax marginal value of reserves increases the profit-maximizing size of reserves.

Referring to Fig. 2b, the downward trend in the tax rate has the same negative intertemporal substitution effect irrespective of the initial tax rate. However, the higher (lower) the initial tax rate and tax deduction, the stronger (weaker) the positive scale effect, as shown by the movement rightward along the flatter extraction line, and thus, current extraction is higher (lower) compared with laissez-faire $\left({E}_{0}^{{\tau }_{H}}>{E}_{0}^{LF}>{E}_{0}^{{\tau }_{L}}\right)$. The higher tax deduction for reserves relative to the higher tax on income increases the total amount to be extracted, whereby present extraction increases despite the incentive to slow extraction. A weak green paradox therefore arises for ${\tau }_{H}$.

### Strong green paradox

Referring to the appendix, a strong green paradox occurs if

20 $$\frac{\left(1-\tau_0\right)}{\underbrace{\left(1-\delta\tau_0\right)}_{(i)}}>\underbrace{\left(\frac{\eta r-\pi}{\eta\left(r-x\right)-\pi}\right)^\alpha}_{(ii)}\underbrace{\left(\frac{\rho+\eta\left(r-x\right)-\pi}{\rho+\eta r-\pi}\right)^{\frac1\eta+\alpha}}_{(iii)}$$

where $\left[\left(\rho +\eta \left(r-x\right)-\pi \right)/\left(\rho +\eta r-\pi \right)\right]<1$ when $x>0$. Term $(i)$ reflects the increase in reserves due to the producer subsidy $\delta >1$. Aggregate emissions increase as reserves and thus the aggregate amount of fossil fuel extracted for sale and combustion increases. Term $(ii)$ reflects the delay of extraction to the future due to the downward trend in the tax rate $x>0$. Delayed emissions cause less NPV damages than earlier emissions. Term $(iii)$ reflects the fact that NPV damages accumulate as emissions increase with fossil fuel burned and as the marginal damages per emissions increase at the rate $\sigma$ where $\rho =r-\sigma$.

Referring to (20), $(iii)<1$ has two implications. First, if the inequality condition for the weak green paradox holds, then the inequality condition for the strong green paradox holds where $(i)>(ii)\Rightarrow$ $(i)>(ii)\times (iii)$. When both initial extraction and reserves are higher under the policy scenario, NPV damages are higher due to the rise in earlier and aggregate emissions. Accordingly, the following policy discussion focuses on parameter values for which the weak green paradox is avoided. Second, the reverse inequality may hold in which case the strong green paradox is avoided where $(i)<$ $(ii)\times (iii)$. The NPV of cumulative damages are lower under the policy scenario when sufficient fossil fuel is left in the ground for longer and emissions are delayed.

A decrease in ${\tau }_{0}$ increases $\left(i\right)$ when $\delta >1$. The intuition is straightforward. The rise in after-tax initial income relative to after-subsidy cost of reserves increases the profit-maximizing size of reserves and thus cumulative emissions from the aggregate amount of extractable fossil fuel.

A high intertemporal rate of decline in the tax rate ${x}_{H}$ has competing effects on the likelihood of a strong green paradox. Referring to (20), term $(ii)$ is higher. A weak green paradox is avoided when $(i)<$ $(ii)$. However, term $(iii)$ is lower. If ($i)>(ii)\times (iii)$, then a strong green paradox arises. This result is summarized in the following remark.

#### Remark 2

*For a sufficiently high rate of decline in the tax rate* $\left({x}_{H}\right)$ *, the weak green paradox is avoided although the strong green paradox arises.*

Intuitively, the stronger negative intertemporal substitution effect of lower future tax rates reduces earlier extraction for sale and delayed emissions cause less NPV damages. However, a steep downward profile for the tax rate increases the profitability of developing more reserves to be extracted in total and higher aggregate emissions raise NPV damages. A strong green paradox arises when the effect of higher aggregate emissions more than offsets the effect of delayed emissions.

Setting $\delta =1$ would mean that the fossil fuel producer faces the same tax treatment as other companies able to claim tax deductions for upfront costs at the initial tax rate. The initial level of the tax rate ${\tau }_{0}$ does not affect the profit-maximizing size of reserves because the tax deduction for upfront costs eliminates the initial tax burden. Referring to (17), a weak green paradox is avoided where $(i)=1\Rightarrow (i)<(ii)$ when $x>0$. However, the effect on (20) is ambiguous. If $1>(ii)\times (iii)$, then a strong green paradox arises. However, if $1<(ii)\times (iii)$ then a strong green paradox is avoided. This result is summarized in the following remark.

#### Remark 3

*Removal of the fossil fuel tax subsidy* $\left(\delta =1\right)$ *avoids the weak green paradox for any downward trend in the tax rate *$\left(x>0\right)$* although the effect on the strong green paradox is ambiguous.*

The intuition for the avoidance of a weak green paradox lies in the condition for profit-maximizing reserves. The initial level of the tax rate reduces the marginal value and marginal cost of reserves by the same amount. Lower future tax rates increase the marginal value of reserves where the marginal cost of reserves increases with the size of reserves. The positive scale effect is therefore weaker than the negative intertemporal substitution of lower future tax rates and initial extraction for sale decreases relative to the laissez-faire scenario.

However, lower future tax rates have an ambiguous effect on the NPV damages of cumulative damages from climate change. On the one hand, the reduction in earlier extraction and the delay of emissions cause less NPV damages. On the other hand, the profitability of developing more reserves increases aggregate emissions and thus NPV damages. The increase in reserves is smaller in the absence of the producer subsidy. Thus, a strong green paradox could be avoided if the effect on NPV damages of delayed emissions is stronger.

A downward trend in the tax rate $\left(x>0\right)$ is more likely to generate a strong green paradox, the higher the shadow value of emissions $\left(\sigma \right)$, and the higher the price elasticity of demand $\left(\eta \right)$. An increase in $\sigma$ and $\eta$ decreases $\left(iii\right)$ when $x>0$. The higher $\sigma$ means that the timing of emissions is less relevant for climate damages. Accordingly, the delay of extraction and emissions due to the downward tax trend is less helpful for the climate. The intuition for the effect of an increase in $\eta$ lies in the tax-adjusted Hotelling rule $\dot{p}/p+x=r$. The higher $\eta$ means higher appreciation in the price for fossil fuel left in the ground as the stock is depleted over time. By slowing depletion, the downward tax trend attenuates this effect and thus NPV damages are less likely to be lower than under the laissez-faire outcome.

It is worth noting what happens in the model if the tax rate increases over time $(x<0)$. Referring to Eq. (17), if $x<0$ then the inequality holds for $\delta >1$ and thus, accounting for subsidized reserves development unambiguously introduces a weak green paradox. Intuitively, the positive intertemporal substitution effect of faster extraction due to higher future tax rates reinforces the positive scale effect of additional reserves due to upfront tax deductions for the cost of reserves development. Referring to Eq. (20), if $x<0$ then term $\left(ii\right)<1$ reflects earlier extraction due to higher future tax rates and term $\left(iii\right)>1$ reflects the reduced profitability of reserves due to the upward-sloping income tax profile. Without the subsidy, the overall effect on NPV damages is ambiguous. However, the strong green paradox arises under subsidized reserves development due to the increase in reserves and thus cumulative damages.

## Quantitative examples and implications

To demonstrate the relevance of our theoretical results, we provide quantitative examples of weak and strong green paradox outcomes for downward-sloping income tax profiles under subsidized development of global crude oil reserves. Parameter values are based on estimates from recent empirical studies and observed relative magnitudes of global crude oil reserves and extraction.^3^ The first quantitative example demonstrates the relevance of the weak green paradox conditions summarized in Remark 1.

Using parameterized Eqs. (14) and (15), we simulate current extraction ${E}_{0}$ and reserves ${S}_{0}$ under laissez-faire $\delta ={\tau }_{0}=0$ and under subsidized reserves development $\delta =1.2$ with different income tax rate levels ${\tau }_{0}$ and intertemporal rates of decline $x>0$. Table 1 quantifies the competing effects of downward-sloping income tax profiles on the level of reserves and the extraction rate. The final column shows the overall effect on the level of current extraction relative to laissez-faire. For ${\tau }_{0}=0.35$ and $x=0.0055 (0.55\%)$, the positive scale effect counterbalances the negative substitution effect leaving the level of current extraction unchanged relative to laissez-faire. The weak green paradox occurs for higher level income tax profiles ${\tau }_{0}=0.45$ which strengthen the positive scale effect of upfront tax deductions for reserves development costs and flatter income tax profiles $x=0.001(0.1\%)$ which weaken the negative intertemporal substitution effect.

**Table 1:** Simulated effect of decreasing income tax profiles on global crude oil reserves and extraction in 2020

Scenario	Rate of tax decline [%]	Reserves [bbl]	Extraction [bbl]	Extraction rate [%]	Extraction relative to LF [%]	Weak Green Paradox (WGP)
Laissez-faire (LF)*	0.00	1694.57	33.72	2.0	0.0	n.a
Tax decline to avoid WGP	0.55	1832.36	33.72	1.8	0.0	No
Low rate of tax decline ${x}_{L}$	0.10	1749.47	34.34	2.0	1.8	Yes
High rate of tax decline ${x}_{H}$	1.00	1923.48	33.09	1.7	− 1.9	No
Low tax rate level ${\tau }_{0}=0.25$	0.55	1816.47	33.43	1.8	− 0.9	No
High tax rate level ${\tau }_{0}=0.45$	0.55	1855.49	34.15	1.8	1.3	Yes

^*^Equivalent to scenario of ${\tau }_{0}=0.35$ with $\delta =1$ and $x=0$

Although steeper income tax profiles $x=0.01(1\%)$ avoid the weak green paradox under subsidized reserves development $\delta >1$, the increase in global oil reserves to 1923.5 billion barrels (bbl) is considerable. If $\delta =1$, then any $x>0$ reverses the inequality in Eq. (17) and the weak green paradox is avoided, whereas the effect on the inequality in Eq. (20) and the strong green paradox is ambiguous. We therefore provide a numerical illustration of the strong green paradox conditions summarized in Remark 3.

**Fig. 3:**
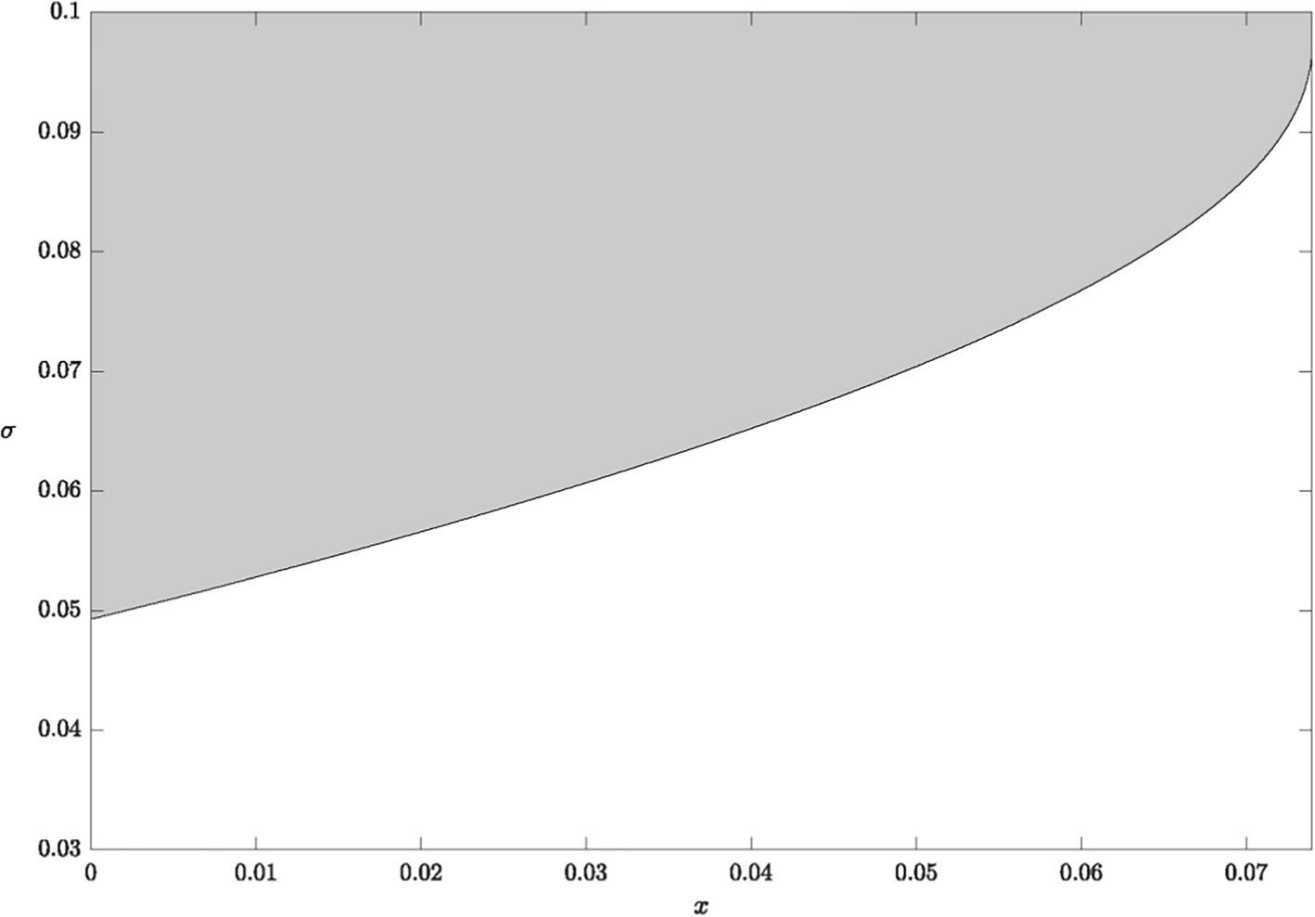
Values of $\sigma$ and $x\in \left(0,r-\frac{\pi }{\eta }\right)$ satisfying strong green paradox conditions (shaded area) when $\delta =1$

Figure 3 illustrates the sensitivity of the strong green paradox condition to growth in marginal damages per emissions over time $\sigma$. We set $\delta =1$ and plot possible values of $\sigma$ and $x>0$ for parameterized Eqs. (17) and (20). The shaded area above the upward-sloping curve depicts values of $\sigma$ and $x>0$ for which the strong green paradox occurs. For example, if $x=0.01(1\%)$ then the strong green paradox occurs for $\sigma >0.053$. The curve is upward sloping because higher values of $x$ delay extraction, while higher values of $\sigma$ imply that the associated delay of emissions is less relevant for damages.

The assumption that earlier emissions cause higher NPV damages than delayed emissions $(\sigma <r)$ does not exclude the possibility of irreversible climate change (Gerlagh 2011). We may reasonably expect that real climate systems feature non-linearities and tipping points where earlier extraction and emissions increase the severity of damages, although the timing of emissions might become secondary to the effect of cumulative emissions if all reserves are extracted. Moreover, crude oil, natural gas, and coal differ regarding reserves in the ground and carbon content per unit of energy. It is worth considering how specified climate dynamics and damage functions for different Representative Concentration Pathway (RCP) scenarios and fossil fuels may affect our quantitative illustration.

Recent calibrated models specify damage functions that incorporate carbon cycle and global temperature changes to quantify the impact of carbon tax profiles and trade-related questions. Cruz and Rossi-Hansberg (2021) estimate regional damage functions for CO_2_ emissions from aggregate coal, gas, and oil under the RCP 8.5 high damages scenario and find that carbon taxes delay rather than reduce total use of carbon. Kotlikoff et al. (2021) incorporate intergenerational and regional distributions to calibrate a damage function that admits winners and losers while approximating aggregate DICE-2016 damages for different RCP scenarios. For the high damages scenario, the optimal policy comprises an increasing carbon tax that materially shortens the interval over which fossil fuels continue to be used, although China’s failure to participate or postponing the carbon tax until 2040 reduces the welfare gain.

The findings of this recent literature suggest that the effect of intertemporal tax profiles on the timing of emissions is secondary to the effect on cumulative emissions for high damages. As coal is more abundant and carbon-intensive, extraction is driven by costs rather than scarcity rents where the increase in cumulative emissions due to lower future tax rates would be more relevant than timing of emissions for damages. Referring to Fig. 3, this implies an increase in the value of $\sigma$ and thus likelihood of the strong green paradox for downward-sloping income tax profiles without subsidized reserves development.

The assumption of exhaustibility remains reasonable for crude oil and natural gas where scarcity rents influence the decision of when to extract. However, the marginal cost of reserves development is less steep for natural gas (Rogner et al. 2012). Referring to Fig. 3, the curve’s vertical intercept decreases as $\alpha$ decreases, which increases the shaded area and thus the likelihood that the strong green paradox occurs at lower values of $\sigma$ where delayed emissions are more relevant for damages.

For a multi-country, multi-industry quantitative trade model with carbon leakage, Farrokhi and Lashkaripour (2021) estimate that unilateral carbon border taxes achieve at most 1% of the reduction in CO_2_ emissions attainable under global cooperation. This highlights the relevance of crude oil in the quantitative examples from our model. While coal emits by far the most carbon dioxide per unit of energy, oil and natural gas received 55% of explicit fossil fuel subsidies in 2020 (Parry et al. 2021). Compared with global cooperation on carbon taxation, agreement on supply-side climate policy could be more successful due to fewer oil producers and the positive terms of trade effect from reducing supply.

## Conclusions

Fossil fuel producers hold more stock to be extracted and burned than would be consistent with global climate change targets. Despite policy commitments to reduce fossil fuel demand and producer subsidies, new reserves continue to be developed. Setting the income tax rate to decline over time is a supply-side climate policy aimed at encouraging producers to leave reserves in the soil for longer, whereby delayed emissions cause less damage than earlier emissions.

The model presented in this paper shows that accounting for upfront tax deductions for reserves development costs induces a perverse outcome where downward-sloping income tax profiles could exacerbate rather than mitigate climate change damages. The weak and strong green paradox describes an unintended rise in contemporaneous emissions and net present value of cumulative damages relative to laissez-faire extraction. The analysis recognizes that the development of reserves, although undertaken upfront, depends on anticipated income from extraction for sale.

The analysis finds that the weak green paradox:

1. arises under a higher level and flatter intertemporal profile of the income tax rate;
2. vanishes for any downward-sloping income tax profile without the producer subsidy, although the strong green paradox may still arise.

The first result challenges the conventional wisdom that downward-sloping tax profiles avoid green paradox outcomes irrespective of the tax rate level. Higher upfront tax deductions reduce the incremental cost relative to value of developing more reserves, while flatter income tax profiles attenuate the incentive to delay extraction to the future. Overall, the present volume extracted for sale increases.

Accounting for subsidized reserves development, lower income tax rate levels or steeper intertemporal tax profiles are required to avoid a weak green paradox. A quantitative illustration of the model shows that setting an income tax rate of 35% to decline at an intertemporal rate of 1% reduces the present volume of crude oil extracted relative to laissez-faire. However, the rise in reserves is considerable which suggests a strong green paradox due to higher net present value damages from cumulative emissions.

The second result implies that if the fossil fuel producer claims tax deductions for the cost of reserves as per any other company at the income tax rate level, then downward-sloping tax profiles raise the value of fossil fuel in the soil which encourages both the development of new reserves and the delay of extraction. The increasing marginal cost of reserves attenuates the former effect whereby the weak green paradox vanishes. The relative importance of delayed emissions to the net present value of cumulative damages determines whether the strong green paradox is also avoided.

Our quantitative illustration shows that the strong green paradox is more likely to occur when growth in marginal damages per emissions over time is high, where delayed emissions are less relevant for the net present value of cumulative damages. This is consistent with the findings of recent calibrated models with specified damage functions which suggest that the effect of intertemporal tax profiles on the timing of emissions is secondary to the effect on cumulative emissions for high damage scenarios.

## Supplementary Information

Below is the link to the electronic supplementary material.

Supplementary file1 (DOCX 167 KB)

## Data Availability

Not applicable.

## Footnotes

1 To illustrate, if ${\tau }_{0}=0.3$ and $\delta =1$, then total tax would be $0.3\left(p\left({E}_{0},{Z}_{0}\right){E}_{0}-\Phi \left({S}_{0}\right)\right)$ or 30 cents per dollar of profit earned at time $t=0$. If $\delta >1$, then fossil fuel developed is subsidized by reducing total tax to $0.3p\left({E}_{0},{Z}_{0}\right){E}_{0}-\delta 0.3\Phi \left({S}_{0}\right)$, which is less than 30 cents per dollar of profit.

2 The mathematical appendix can be found in the Electronic Supplementary Material.

3 The parameter values are explained in the Electronic Supplementary Material.
