# Supplementary material for: The supply-side climate policy of decreasing fossil fuel tax profiles: can subsidized reserves induce a green paradox?
Source: Clim Change. 2022 Aug 22;173(3-4):27. doi: 10.1007/s10584-022-03389-w (PMC9395873; doi:10.1007/s10584-022-03389-w)
Supplement: Supplementary file 1 — Supplementary file1 (DOCX 167 KB) [file 10584_2022_3389_MOESM1_ESM.docx]

**Supplementary Material for Article Submitted to Climatic Change**

Appendix A: Supplementary material on fossil fuel producer's optimal control problem

The current valued Hamiltonian is

$$\hat{H}=\left( 1-\tau_{t} \right)p_{t}E_{t}+\mu_{t}\left( -E_{t} \right)$$

where the control variable is $E_{t}$ and the state variable is $S_{t}$ .

The necessary first order and transversality conditions for an optimal solution are

$$\begin{aligned} \hat{H}_{E_{t}}=0\Rightarrow\left( 1-\tau_{t} \right)p_{t}=\mu_{t} \#\left( A1.1 \right) \end{aligned}$$

$$\begin{aligned} \dot{\mu}-r\mu=-\hat{H}_{S}\Rightarrow\dot{\mu}=r\mu_{t} \#\left( A1.2 \right) \end{aligned}$$

$$\begin{aligned} \mu_{0}=\left( 1-\delta\tau_{0} \right)\Phi^{'}\left( S_{0} \right) \#\left( A1.3 \right) \end{aligned}$$

$$\begin{aligned} \lim_{t\to\infty} e^{-rt}\mu_{t}S_{t}=0 \#\left( A1.4 \right) \end{aligned}$$

where taking the time derivative of (A1.1) and substituting from (A1.2) gives equation (8)

$$\begin{aligned} -\frac{\dot{\tau}}{\left( 1-\tau\right)}+\frac{\dot{p}}{p}=r \#\left( A1.5 \right) \end{aligned}$$

where $-\dot{\tau}/\left( 1-\tau\right)=x$.

The general solution to (A1.2) is

$$\begin{aligned} \mu_{t}=C_{1}e^{rt} \#\left( A1.6 \right) \end{aligned}$$

where $C_{1}$ is the constant of integration. For constant price elasticity demand, $p_{t}=\left( Z_{t} \right)^{1/\eta}\left( E_{t} \right)^{-1/\eta}$ and $\eta\equiv-\frac{dE_{t}}{E_{t}}/\frac{dp_{t}}{p_{t}}$.

From (A1.1) and (A1.6)

$$\begin{aligned} \left( 1-\tau_{t} \right)p_{t}=\left( 1-\tau_{0} \right){Z_{0}}^{\frac{1}{\eta}}e^{\left( \frac{\pi}{\eta}+x \right)t}\left( E_{t} \right)^{-\frac{1}{\eta}}=C_{1}e^{rt} \#\left( A1.7 \right) \end{aligned}$$

where substituting from (1) and integrating gives the general solution

$$\begin{aligned} S_{t}=\left[ \frac{\left( 1-\tau_{0} \right)}{C_{1}}\left( \frac{Z_{0}}{\eta\left( r-x \right)-\pi} \right)^{1/\eta} \right]^{\eta}e^{-\left[ \eta\left( r-x \right)-\pi\right]t}+C_{2} \#\left( A1.8 \right) \end{aligned}$$

where $C_{2}$ is the constant of integration. The boundary condition $\lim_{t\to\infty}S(t)=0$, noting $\eta\left( r-x \right)-\pi>0$, gives $C_{2}=0$.

For fossil fuel development cost $\Phi\left( S_{0} \right)=\varphi\frac{{S_{0}}^{1+\alpha}}{1+\alpha}$ where $\alpha>0$, from (A1.3)

$$\begin{aligned} C_{1}=\mu_{0}=\left( 1-\delta\tau_{0} \right)\varphi{S_{0}}^{\alpha} \#\left( A1.9 \right) \end{aligned}$$

which substituting in (A1.8) gives

$$\begin{aligned} S_{0}=\left[ \frac{\left( 1-\tau_{0} \right)}{\left( 1-\delta\tau_{0} \right)\varphi}\left( \frac{Z_{0}}{\eta\left( r-x \right)-\pi} \right)^{\frac{1}{\eta}} \right]^{\frac{\eta}{1+\alpha\eta}} \#\left( A1.10 \right) \end{aligned}$$

From (A1.9) and (A1.10), the particular solution for (A1.6) is therefore

$$\mu_{t}=\left( 1-\tau_{0} \right)\left( \frac{Z_{0}}{S_{0}\left( \eta\left( r-x \right)-\pi\right)} \right)^{\frac{1}{\eta}}e^{rt}$$

and, using (A1.7) and (A1.8), the optimal paths for $p_{t}$ and $E_{t}$ are

$$p_{t}=\left( \frac{Z_{0}}{S_{0}\left( \eta\left( r-x \right)-\pi\right)} \right)^{\frac{1}{\eta}}e^{\left( r-x \right)t}$$

$$E_{t}={S_{0}\left( \eta\left( r-x \right)-\pi\right)e}^{-\left( \eta\left( r-x \right)-\pi\right)t}$$

$$S_{t}=S_{0}e^{-\left( \eta\left( r-x \right)-\pi\right)t}$$

where $S_{0}$ is given by (A1.10).

Setting $\tau_{0}=x=0$, the laissez-faire $\left( LF \right)$ solution is

$$\mu_{t}^{LF}=\left( \frac{Z_{0}}{S_{0}^{LF}\left( \eta r-\pi\right)} \right)^{\frac{1}{\eta}}e^{rt}$$

$$p_{t}^{LF}=\left( \frac{Z_{0}}{S_{0}^{LF}\left( \eta r-\pi\right)} \right)^{\frac{1}{\eta}}e^{rt}$$

$$E_{t}^{LF}=S_{0}^{LF}\left( \eta r-\pi\right)e^{-\left( \eta r-\pi\right)t}$$

$$S_{0}^{LF}=\left[ \frac{1}{\varphi}\left( \frac{Z_{0}}{\eta r-\pi} \right)^{1/\eta} \right]^{\frac{\eta}{1+\alpha\eta}}$$

Green paradox

A weak green paradox occurs when income taxation raises current emissions:

$$E_{0}>E_{0}^{LF}$$

$$S_{0}\left( \eta\left( r-x \right)-\pi\right)>S_{0}^{LF}\left( \eta r-\pi\right)$$

$$\left( \eta\left( r-x \right)-\pi\right)^{1-\frac{1}{1+\alpha\eta}}\left[ \frac{\left( 1-\tau_{0} \right)}{\left( 1-\delta\tau_{0} \right)} \right]^{\frac{\eta}{1+\alpha\eta}}>\left( \eta r-\pi\right)^{1-\frac{1}{1+\alpha\eta}}$$

$$\frac{\left( 1-\tau_{0} \right)}{\left( 1-\delta\tau_{0} \right)}>\left[ \frac{\eta r-\pi}{\eta\left( r-x \right)-\pi} \right]^{\alpha}$$

where $\delta>1\Rightarrow\frac{\left( 1-\tau_{0} \right)}{\left( 1-\delta\tau_{0} \right)}>1$ and $x>0\Rightarrow\left[ \frac{\eta r-\pi}{\eta\left( r-x \right)-\pi} \right]^{\alpha}>1$.

Substituting for $S_{0}$ and $S_{0}^{LF}$ in $E_{t}$ and $E_{t}^{LF}$ gives

$$E_{t}=\left[ \frac{\left( \eta\left( r-x \right)-\pi\right)^{\alpha}\left( 1-\tau_{0} \right){Z_{0}}^{\frac{1}{\eta}}}{\left( 1-\delta\tau_{0} \right)\varphi} \right]^{\frac{\eta}{1+\alpha\eta}}e^{-\left( \eta\left( r-x \right)-\pi\right)t}$$

$$E_{t}^{LF}=\left[ \frac{\left( \eta r-\pi\right)^{\alpha}{Z_{0}}^{\frac{1}{\eta}}}{\varphi} \right]^{\frac{\eta}{1+\alpha\eta}}e^{-\left( \eta r-\pi\right)t}$$

The NPV climate damages, noting $\rho-\sigma>0$ and $\eta\left( r-x \right)-\pi>0$, are

$$D=\int_{0}^{\infty} e^{-\rho t}\theta_{0}E_{t}dt$$

$$=\int_{0}^{\infty} e^{-\left( \rho+\eta\left( r-x \right)-\pi\right)t}\theta_{0}\left[ \frac{\left( \eta\left( r-x \right)-\pi\right)^{\alpha}\left( 1-\tau_{0} \right){Z_{0}}^{\frac{1}{\eta}}}{\left( 1-\delta\tau_{0} \right)\varphi} \right]^{\frac{\eta}{1+\alpha\eta}}dt$$

$$=\left. \frac{-1}{\left( \rho+\eta\left( r-x \right)-\pi\right)} \right|_{0}^{\infty}e^{-\left( \rho+\eta\left( r-x \right)-\pi\right)t}\theta_{0}\left[ . \right]^{\frac{\eta}{1+\alpha\eta}}$$

$$=\frac{\theta_{0}}{\left( \rho+\eta\left( r-x \right)-\pi\right)}\left[ \frac{\left( \eta\left( r-x \right)-\pi\right)^{\alpha}\left( 1-\tau_{0} \right){Z_{0}}^{\frac{1}{\eta}}}{\left( 1-\delta\tau_{0} \right)\varphi} \right]^{\frac{\eta}{1+\alpha\eta}}$$

$$D^{LF}=\int_{0}^{\infty} e^{-\rho t}\theta_{0}E_{t}^{LF}dt$$

$$=\frac{\theta_{0}}{\left[ \rho+\eta r-\pi\right]}\left[ \frac{\left( \eta r-\pi\right)^{\alpha}{Z_{0}}^{\frac{1}{\eta}}}{\varphi} \right]^{\frac{\eta}{1+\alpha\eta}}$$

A strong green paradox occurs when income taxation raises NPV damages:

$$D>D^{LF}$$

$$\frac{\left( \eta\left( r-x \right)-\pi\right)^{\frac{\alpha\eta}{1+\alpha\eta}}}{\rho+\eta\left( r-x \right)-\pi}\left[ \frac{\left( 1-\tau_{0} \right)}{\left( 1-\delta\tau_{0} \right)} \right]^{\frac{\eta}{1+\alpha\eta}}>\frac{\left( \eta r-\pi\right)^{\frac{\alpha\eta}{1+\alpha\eta}}}{\rho+\eta r-\pi}$$

$$\left[ \frac{\left( 1-\tau_{0} \right)}{\left( 1-\delta\tau_{0} \right)} \right]^{\frac{\eta}{1+\alpha\eta}}>\left( \frac{\eta r-\pi}{\eta\left( r-x \right)-\pi} \right)^{\frac{\alpha\eta}{1+\alpha\eta}}\frac{\rho+\eta\left( r-x \right)-\pi}{\rho+\eta r-\pi}$$

$$\frac{\left( 1-\tau_{0} \right)}{\left( 1-\delta\tau_{0} \right)}>\left( \frac{\eta r-\pi}{\eta\left( r-x \right)-\pi} \right)^{\alpha}\left[ 1-\frac{\eta x}{\rho+\eta r-\pi} \right]^{\frac{1}{\eta}+\alpha}$$

**Appendix B: Supplementary material on parameter values for quantitative examples**

Parameter values for the cost of reserves development function are chosen to fit Global Energy Assessment (GEA) estimates of the global oil marginal cost curve for an aggregate of 18 GEA regions in 2007 (Rogner et al 2012). The figure below plots the potential reserves from conventional and non-conventional oil resources against the marginal cost of development as reserves and displays the trend line fitted to a power function. The fitted marginal cost curve is increasing and convex in the size of reserves and provides estimated values of $\varphi=0.14$ and $\alpha=1.46$.


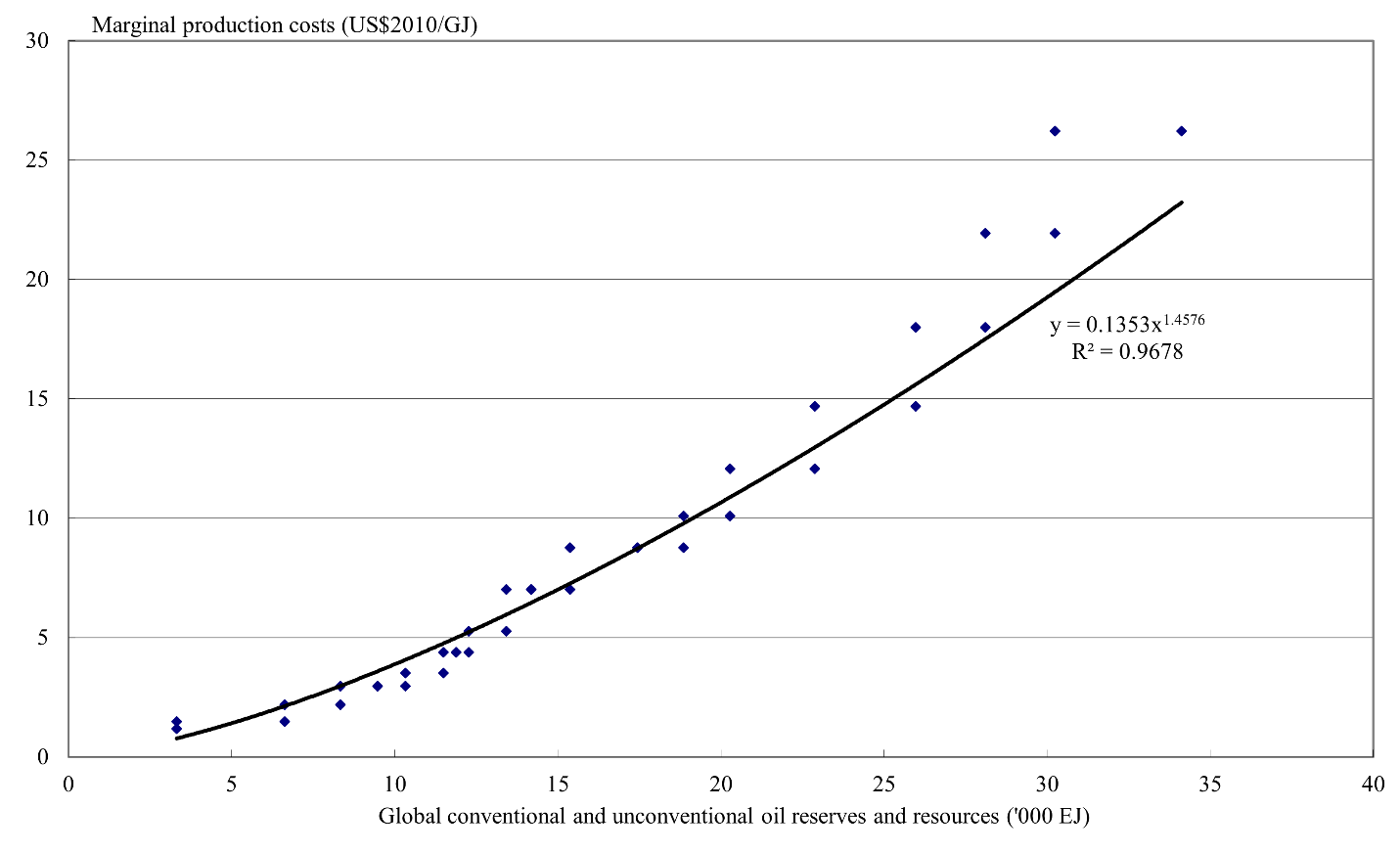


**Fig. B.1** Parameter values for marginal cost of oil reserves development

The model’s predicted extraction rate without downward-sloping income tax profiles, $\eta r-\pi=0.02$, corresponds to the average annual extraction rate of global crude oil, 2000-2020 (British Petroleum, 2021). We set the discount rate on future revenue from developed oil reserves at $r=0.1$, as commonly assumed in the academic literature (Anderson et al. 2018, Erickson et al. 2020).^[[1]](#footnote-1)^ The intertemporal rate of change in demand $\pi=0.007$ is proxied by the average annual rate of forecast growth in global crude oil consumption, 2020 to 2045 (OPEC 2021). The numerical value of $\eta=0.27$ is an average of recent global price elasticity of oil demand estimates, $0.26$ (Kilian 2020) and $0.28$ (Herrera and Rangaraju 2020).

The income tax rate level $\tau_{0}=0.35$ is a mid-range estimate of effective US federal and foreign income tax rates for oil companies of 0.25 and 0.45, respectively (TCS, 2014). Setting $\delta=1.2$ uplifts the rate of tax deductions for reserves development costs to ${\delta\tau}_{0}=0.42$, as exemplified by the intangible drilling cost (IDC) subsidy in the United States. Erickson et al (2020) estimate that the IDC subsidy, valued on a present value basis, reduces the breakeven oil price of new projects by $4.20 to $7.30 per barrel in 2016 US dollars. Assuming an effective US tax rate of 0.25, the equivalent reduction in breakeven price for $\tau_{0}=0.35$ ranges from $5.88 to $10.22. Using the 2016 crude oil price of $43.14 per barrel (International Monetary Fund, 2022) as an upper bound for the breakeven price, the corresponding value of $\delta$ ranges from 1.14 to 1.24 with an average of 1.2. The remaining parameter value $Z_{0}=366.58$ calibrates simulated reserves under subsidized reserves development with $\tau_{0}=0.35$ and $x=0$ to global crude oil reserves in 2020 of 1732.4 billion barrels (British Petroleum, 2021) for illustrative purposes.

**References**

Anderson S, Kellogg R, Salant S (2018) Hotelling under pressure. J Polit Econ 126: 984-1026

British Petroleum (2021) British Petroleum BP Statistical review of world energy <https://www.bp.com/en/global/corporate/energy-economics/statistical-review-of-world-energy.html>

Erickson P, van Asselt, H, Koplow D, Lazarus M, Newell P, Oreskes N, Supran, G (2020) Why fossil fuel producer subsidies matter. Nature 578: E1-4

Herrera J, Rangaraju S (2020) The effect of oil supply shocks on US economic activity: What have we learned? J Appl Econom 35: 141-159

International Monetary Fund (2022) Spot crude oil price of West Texas Intermediate, Washington DC, retrieved from FRED, Federal Reserve Bank of St. Louis. <https://fred.stlouisfed.org/series/WTISPLC>

Kilian L (2020) Understanding the estimation of oil demand and oil supply elasticities. Federal Reserve Bank of Dallas Working Paper 2027, September 2020, Dallas

Metcalf G (2018) The impact of removing tax preferences for US oil and natural gas production: Measuring tax subsidies by an equivalent impact approach. J Assoc Environ Res Econ 5: 1-37

OPEC (2021) Organization of Petroleum Exporting Countries, World Oil Outlook 2045, Vienna. <https://woo.opec.org/pdf-download/>

Rogner H, Aguilera R, Archer C, Bertani R, Bhattacharya S, Dusseault M (2012) Chapter 7: Energy resources and potentials. In: Zou J (ed) Global energy assessment – toward a sustainable future. Cambridge University Press, Cambridge, pp 425-512

TCS (2014) Taxpayers for Common Sense, Effective tax rates for oil and gas companies cashing in on special treatment July 2014, Washington DC

van der Ploeg F, Rezai A (2020) The risk of policy tripping and stranded carbon assets. J Environ Econ Manag 100: Article 102258

1. This discount rate approximates the real interest rate $(0.04)$ plus depreciation rate $(0.05)$ assumed in van der Ploeg and Rezai (2020) and is less than the discount rate of $0.15$ assumed in industry studies (Metcalf 2018). [↑](#footnote-ref-1)
